# A Novel Workflow for Electrophysiology Studies in Patients with Brugada Syndrome

**DOI:** 10.3390/s24134342

**Published:** 2024-07-04

**Authors:** Valentina Hartwig, Maria Sole Morelli, Nicola Martini, Paolo Seghetti, Davide Tirabasso, Vincenzo Positano, Sara Latrofa, Giacomo Mansi, Andrea Rossi, Alberto Giannoni, Alessandro Tognetti, Nicola Vanello

**Affiliations:** 1 Institute of Clinical Physiology (IFC), 56124 Pisa, Italy; 2 Fondazione Toscana Gabriele Monasterio, 56124 Pisa, Italypositano@ftgm.it (V.P.);; 3 Health Science Interdisciplinary Center, Scuola Superiore Sant’Anna, 56127 Pisa, Italy; 4 Dipartimento di Ingegneria dell’Informazione, University of Pisa, 56124 Pisa, Italy; 5 Department of Surgical, Medical and Molecular Pathology and Critical Care Medicine, University of Pisa, 56124 Pisa, Italy; 6 Research Center “E. Piaggio”, University of Pisa, 56124 Pisa, Italy

**Keywords:** endocardial signals processing, Brugada Syndrome (BrS), OpenEP, multi-parametric mapping

## Abstract

Brugada Syndrome (BrS) is a primary electrical epicardial disease characterized by ST-segment elevation followed by a negative T-wave in the right precordial leads on the surface electrocardiogram (ECG), also known as the ‘type 1’ ECG pattern. The risk stratification of asymptomatic individuals with spontaneous type 1 ECG pattern remains challenging. Clinical and electrocardiographic prognostic markers are known. As none of these predictors alone is highly reliable in terms of arrhythmic prognosis, several multi-factor risk scores have been proposed for this purpose. This article presents a new workflow for processing endocardial signals acquired with high-density RV electro-anatomical mapping (HDEAM) from BrS patients. The workflow, which relies solely on Matlab software, calculates various electrical parameters and creates multi-parametric maps of the right ventricle. The workflow, but it has already been employed in several research studies involving patients carried out by our group, showing its potential positive impact in clinical studies. Here, we will provide a technical description of its functionalities, along with the results obtained on a BrS patient who underwent an endocardial HDEAM.

## 1. Introduction

Patients with Brugada Syndrome (BrS) exhibit a ST-segment elevation followed by a negative T-wave in the right precordial leads (V1-V2) on the surface electrocardiogram (ECG), also known as the ‘type 1’ ECG pattern [1,2]. BrS patients have an increased risk of sudden cardiac death (SCD) due to polymorphic ventricular arrhythmias tachycardia (VT) [1,2]. Typical symptoms of BrS include syncope, agonal nocturnal respiration, palpitation, and sudden cardiac death (SCD), usually occurring between 38 and 48 years of age [3]. However, it is important to note that most BrS patients are asymptomatic. Up to 0.5–1% of asymptomatic BrS patients experience an arrhythmic event each year [4,5,6], thus, risk stratification in this cohort of patients is crucial.

The causes of VT in BrS patients are still uncertain and the subject of much debate. Recent studies have identified a critical area on the right ventricular outflow tract (RVOT) surface epicardium that harbors fractionated electrograms and slow conduction, which are likely responsible for the electrocardiographic manifestations of BrS.

It has been suggested that the typical ECG phenotype of BrS patients may be generated by the presence of both genetic mutations and cardiac structural changes, which may also lead to ventricular arrhythmias in some cases [7]. These alterations are mainly explained by two prevailing theories: the hypothesis of altered repolarization and altered depolarization. The first hypothesis [8,9] suggests that there is a difference in the duration of action potentials in the RVOT due to increase of repolarizing currents between the epicardium and endocardium. The increase in repolarizing currents results in two main effects: the formation of a transmural voltage gradient and the triggering of arrhythmogenic mechanisms dictated by phase 2 re-entry circles. These effects produce the type 1 pattern observed in patients who are at risk of experiencing BrS. Experimental models have demonstrated that the transmural dispersion of repolarization is linked to the coved elevation of the ST segment at J-point. Therefore, to assess the presence of a diseased substrate, it is important to investigate the distribution of unipolar electrogram amplitude at J-point relative to the baseline on the map of the right ventricle. The depolarization hypothesis [8,9] suggests that there is slow electrical conduction in the RVOT, causing a delay in the action potential onset when compared to the rest of the right ventricle. When the excitation impulse reaches the right ventricle, the apex is depolarized first, followed by the RVOT. The resulting ECG pattern shows an initial raised ST segment followed by sub-leveling, forming the typical Type 1Brugada pattern. Therefore, studying the distribution of the activation time (AT) parameter on the map of the right ventricle is of interest.

The risk stratification of asymptomatic individuals with spontaneous type 1 ECG pattern remains a topic of debate. Several multi-factor risk scores have been proposed, but none have proven to be effective [10,11,12,13]. In clinical practice, the only widely used tool for risk stratification is inducibility during programmed ventricular stimulation (PVS), although several studies have questioned its sensitivity and specificity in predicting ventricular fibrillation (VF) [5,6,14,15].

Previous studies [4,7,16,17,18] performed high-density electro-anatomical mapping (HDEAM) in BrS patients to gain insight into the pathological substrate. Usually, quantitative parameters are estimated from the patient’s unipolar or bipolar electrograms (e.g., activation time) and the computed parametric map is displayed onto the surface mapped with the catheter. Depending on the parameter, a thresholding of the values displayed may reveal an abnormal region, which should correlate with the presence of pathological substrate.

Right ventricular (RV) HDEAM has revealed the presence of low-voltage areas (LVAs) in the epicardial RVOT of BrS subjects. Recent studies have proposed that endocardial LVAs are located in a corresponding position in the endocardial layer. These LVAs reflect structural myocardial abnormalities [19] and may represent potential targets for treatment in patients with ventricular arrhythmias [20]. Furthermore, in asymptomatic patients with BrS, wider LVAs in the RVOT at unipolar endocardial HDEAM have been linked to histological abnormalities, ventricular arrhythmia inducibility at EPS, and an increased risk of SCD [17,19,20].

A spontaneous diagnostic pattern may indicate the presence of Brugada Syndrome. However, in most cases, it is necessary to use a pharmacological test to rule out the presence of Brugada Syndrome or to confirm the diagnosis. In this type of study, the patient is typically subjected to two HDEAM acquisitions, resulting in the creation of two distinct data structures: pre-infusion (PRE) data and post-infusion (POST) data. The RV surface and the unipolar electrograms with associated acquisition positions are stored in each data structure.

Many studies report data on BrS patients [5,13,17,18,21]; however, each electrophysiology laboratory uses its preferred parameters and acquisition methods, so that a comparison between datasets is oftentimes impossible. Indeed, data are reported as the mean over the whole RV surface or just in the RVOT, or different methods are used to estimate the same parameters, or different parameters are computed altogether.

The aim of this work is to describe a data processing workflow for the endocardial signals acquired with HDEAM from BrS patients. The main objective is to calculate the parameters of interest and create the respective multi-parametric maps of the RV. In addition, some innovative algorithms to process the parametric maps obtained based on the user-defined clinical requirements are provided. These algorithms allow for a thorough analysis of the electrophysiological condition of the right ventricle. This analysis can be useful for both research purposes and for determining a suitable patient-specific treatment plan. Furthermore, this workflow could be used as an initial standardization of further data processing studies.

## 2. Materials and Methods

This section describes the five main steps of the data processing workflow. It begins with the data exported by the electro-anatomical mapping system, followed by the conversion of the data from the proprietary format to a Matlab structure. Next, the features extraction and selection of the region of interest in the RV area are explained. Finally, the creation of 3D multi-parametric maps and data analysis steps are described.

### 2.1. High-Density Right Ventricular Electro-Anatomical Mapping

Endocardial HDEAM is typically performed during sinus rhythm using a three-dimensional (3D) non-fluoroscopic mapping system (e.g., CARTO 3, Biosense-Webster, Diamond Bar, CA, USA). The right ventricular mesh reconstruction can be defined with either the ablation catheter (SmartTouch™^®^ or SmartTouch SF™^®^, Biosense Webster, Diamond Bar, CA, USA) or a multipolar catheter (high-resolution multi-electrode mapping). The DecaNav^®^ catheter, manufactured by Biosense-Webster in Diamond Bar, CA, USA, has ten 1-mm electrodes with a 2-8-2 interelectrode spacing. It can be used with either its 11th electrode as a unipolar reference in the inferior vena cava or the Wilson Central Terminal (WCT) of the surface ECG.

The CARTO 3 system is an advanced imaging technology that employs electromagnetic technology to create three-dimensional (3D) real-time maps of a patient’s heart structures [22]. The system is designed to assist electrophysiologists in navigating the heart by generating an accurate 3D map, in addition to identifying the precise location and orientation of catheters within the heart during diagnostic and therapeutic procedures for patients suffering from heart rhythm conditions (cardiac arrhythmias).

The system functions in a manner analogous to an advanced navigation system in a car, providing the driver with visual and audio cues to ensure the safe parking and operation of the vehicle. During a therapeutic catheter ablation procedure, doctors insert a catheter through a small incision in the groin. The catheter is then guided to the heart through a blood vessel in the leg [23]. Due to the contact of the ablator catheter with the heart surface, it is possible to perform an anatomical reconstruction of the heart itself. Subsequently, an electrical signal can be acquired from the points of interest. The anatomical and electrical 3D map reconstruction allows for the identification of areas that will subsequently be treated by radio frequency ablation with precision.

In order to facilitate the localization of the catheter during the invasive procedure, the CARTO 3 system employs a magnetic location system. This is achieved through the generation of a magnetic field by a location pad fixed under the patient bed.

The catheters are equipped with a passive sensor, which renders them visible within the magnetic field. The sensor provides real-time information, allowing catheter localization (mean accuracy: 1 mm).

The system is capable of extending the magnetic localization system with advanced catheter localization technology, based on high-frequency, low-power current emission from every electrode in each catheter connected to the system. The current of each electrode is measured, and its position is calculated using a proprietary algorithm (mean accuracy: 3 mm).

To ensure accurate mapping, the CARTO 3 system creates a virtual position reference or body coordinate system (BCS). The system utilizes the positions of three back patches, which are related to each other and to the location pad, to determine this initial position reference. The system monitors the location reference to detect any changes during this study. When this study starts, the system records the location of the back patches. From this point onwards, each point or location is determined in relation to the back patches. The CARTO 3 system is designed to continuously monitor the position of the three back patches relative to each other and to the location pad.

There are two main categories of mapping methods: fast anatomical mapping (FAM) and Electro-anatomical mapping (EA). FAM generates anatomical maps by processing a large amount of location data instead of individual location points, while EA builds maps through point-by-point acquisition. When using a focal ablation catheter, each point reflects the position and electrical data of the tip electrode. When using a multi-electrode map (MEM) catheter, the acquired point reflects the location and electrical data of each available mapping electrode. The resulting maps are generated by combining information from cardiac electrograms with the respective location data. The CARTO3 system presents a real-time screen display of a color-coded 3D geometric representation of the cardiac chamber. To view and analyze local activation time, voltage information, and other data, simply select the specific map.

A contact force sensing ablation catheter is used to validate bipolar and unipolar electrograms. To avoid misleading electrogram annotations, points with a contact force less than 5 g or greater than 25 g are discarded. Simultaneous recording and analysis of bipolar (30–500 Hz) and unipolar (1–240 Hz) signals is conducted to determine the amplitude, duration, relation to the surface QRS, and presence of multiple components. Each electro-anatomical point is assigned a unique bipolar and unipolar value. The technique of anatomical right ventricle reconstruction, previously described [17], is employed.

The Carto3 system is the focus of this work. The data exported from the Carto3 system are mainly divided into two blocks: surface data and electrogram data. All individual exported data types representing the geometric and electrical data captured by the system are grouped into these two categories. The data are generally formatted with a series of XML files that describe this study’s characteristics, a set of 12 text files per mapping point that describe the electrogram’s characteristics, and a file that details the chamber’s geometry and the electro-anatomical maps generated during the clinical case.

### 2.2. OpenEP Matlab: Creating Data Structure and Base Functions

Mapping systems are commonly used to guide catheter-based ablation procedures [24]. They serve several essential functions, such as providing anatomical representation and creating electrophysiological maps. The data acquired by these systems offer crucial information on the morphology of the ventricular myocardium and its electrical function. The electro-anatomical mapping platforms interpret this data by creating maps that depict the parameters of interest, such as voltage and activation times, or by visualizing the electrogram. The data exported from these systems is stored in proprietary formats that can be difficult to access and are inefficient in terms of storage space. To begin a more detailed analysis, the mapping data from clinical systems must be exported into a format that is more memory efficient and can be processed easily.

**Figure 1:**
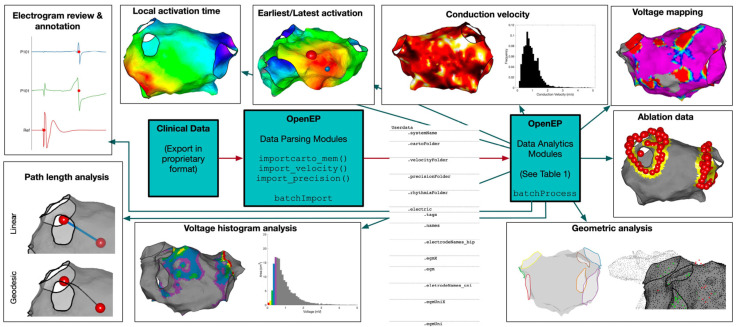
OpenEP Overview. Reproduced from [25].

The OpenEP framework [25], implemented in Matlab, is designed for evaluating electro-anatomical mapping data and providing core functionality for electroanatomic mapping research in a space-efficient and accurate manner. Figure 1 shows an overview of OpenEP framework.

This open-source software allows for the inspection of data exported by electro-anatomical mapping systems commonly used in clinical studies. Currently, OpenEP (Release v1.0.03) has standard implementations for three clinical systems: Carto3 (Biosense-Webster, Diamond Bar, CA, USA), Ensite Velocity (St Jude Medical, St. Paul, MN, USA), and Ensite Precision (Abbot, Plymouth, MN, USA). Following the export of data from one of the aforementioned systems, the processing of a dataset utilizing OpenEP starts with the invocation of an import function. The user is required to select the pertinent study files, the clinical map of interest, the reference mapping channel and an ECG channel. The subsequent dataset analysis is fully automated and involves the creation of a data structure, called userdata, in the workspace, which can also be saved to disk. By using built-in interpolation functions, it is possible to view 3D maps depicting the activation time of the ventricle, tension maps, or other parameters of interest. Furthermore, OpenEP can be employed to facilitate access to electro-anatomical mapping data electrograms. The software also provides tools that can be utilized to identify ablation sites through the use of labels within single-position points. The electro-anatomical points acquired by CARTO3 on the RV surface have location data stored in specific field of the userdata structure. The EgmX field contains the three-dimensional coordinates of the points acquired by the catheter during the electro-anatomical mapping phase, while EgmSurfX contains the three-dimensional coordinates of the same points but projected on the nearest point of the reconstructed virtual surface.

To optimize data storage, OpenEP format employs three techniques. Firstly, OpenEP eliminates redundancy in data, ensuring that there is only one copy of each unique electrogram. Secondly, the format employs a single data structure to store the entire dataset, including anatomical and electrical data, eliminating the file system overload needed to store a large number of files. Finally, data are stored as binary files instead of as a series of text files. In the format exported by clinical mapping systems, each individual patient data set typically has a size of 1–2 GB.

The OpenEP data structure was designed with extensibility in mind. In fact, when creating geometric maps of electrophysiological parameters, such as the electrogram voltage or the activation time, a three-dimensional interpolation is performed, and a visual color representation of the physiological parameter of interest is provided. This interpolation is commonly performed using commercially available clinical electro-anatomical mapping platforms. OpenEP allows access and analysis of these clinical data interpolations. However, there are numerous methods for performing spatial interpolation, which can lead to disparate interpretations of the same data. OpenEP, therefore, provides its own internal framework for executing interpolations based on the electrical data originally acquired. The OpenEP function dedicated to the interpolation algorithm is a key function for performing this task and can be easily modified/extended to use alternative methods for data interpolation.

The OpenEP data format is the foundation of the basic architecture, which also includes the Data Parsing and Data Analytics modules.

When creating geometric maps of electrophysiological parameters, such as electrogram voltage or activation time, OpenEP performs a three-dimensional interpolation and provides a visual color representation of the physiological parameter of interest. The ‘userdata’ structure created by OpenEP allows for the extraction of the following main information: the coordinates of the anatomical points on the RV surface acquired during the FAM, and the coordinates of the points containing electrophysiological information (EA points) sampled on the RV surface during the electro-anatomical mapping.

For each sampled electro-anatomical point, the ‘userdata’ structure contains the following main information: unipolar electrograms, bipolar electrograms, 12-lead surface ECG electrograms, and the force applied with the electrode on the RV surface to acquire electrophysiological information.

### 2.3. Features Extraction and ROI Selection

The analysis of unipolar signals included amplitude, waveform, length, and their relation to the surface QRS. This was performed using homemade software written in Matlab^®^, R2020b (MathWorks, Inc., Natick, MA, USA) which also incorporates the functionality of a Python (Release 1.6.1) toolbox for the implementation of some specific calculations. The following main features were extracted:

#### 2.3.1. Unipolar Electrogram J-Point Elevation (Uni-JEl)

The ECG J-points are automatically calculated for each point map [26] using, in general, V2 ECG derivation. When the quality of V2 derivation is low, it is possible to choose a different ECG derivation to use for J-point calculation. The median J-point is extracted, and the unipolar values at the J-point (Uni-JEl) for each point map are calculated. Uni-JEl values are interpolated on the mesh cells extracted from CARTO3 using the Visualization Toolkit library [27] to create Uni-JEl maps. The electro-anatomical data are represented using a color scale on the anatomy model exported from CARTO3 as a triangulated mesh.

#### 2.3.2. Activation Time (AT)

The activation time (AT) is calculated as the time difference between the minimum of the endocardial unipolar signal derivative and the minimum of the V2 surface lead [28]. The calculation is performed within a time window of interest, which is obtained from two fields of the userdata structure: referenceAnnotation, which specifies the timing reference arbitrarily selected on a recording (intracardiac electrogram or surface ECG lead) representative of activation of the chamber; and window of interest (woi), which refers to the range of activation times surrounding reference activation. Typical values for these fields are 2000 ms (referenceAnnotation) and [−100 ms 100 ms] (woi).

#### 2.3.3. Activation Recovery Interval (ARI)

The activation recovery interval (ARI) is used to estimate action potential duration [29] and has been previously used to evaluate spatial repolarization dispersion in BrS [12,18,21,30,31].

An automated algorithm for ARI calculation using endocardial UEGs in each point of the RV was created using Matlab^®^. The ARI was calculated starting from the minimum dV/dt of the local unipolar QRS to the maximum dV/dt of the following local T wave, as previously described by Wyatt et al. [29]. If there was noise, a premature ventricular beat, or abnormal T-wave morphologies present, the UEG was discarded.

#### 2.3.4. RV 3D Map Creation and ROI Selection

**Figure 2:**
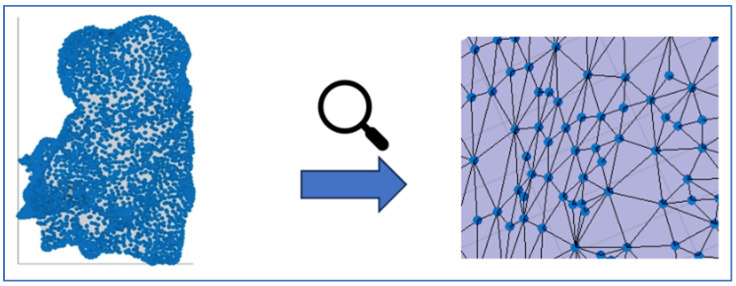
RV 3D virtual surface with mesh triangulation.

The right ventricle virtual surface can be extracted by accessing the ‘userdata’ structure. This surface is reconstructed through triangulation, along with other associated information. The points of the 3D virtual surface can be represented by the vertices of the triangles (Figure 2). These vertices can then be used to interpolate the sampled electro-anatomical points onto the virtual surface generated by anatomical mapping. This creates a 3D map of the RV.

Among the various information available, we can extract the following: the vertices of the triangles (rPts) representing the points of the 3D virtual surface (hSurf); the coordinates relative to the triangles’ centroids; the triangles’ area (mm^2^). The acquired point coordinates and their associated parameter value are then inserted into the tempts matrix and the tempdata vector, respectively.

**Figure 3:**
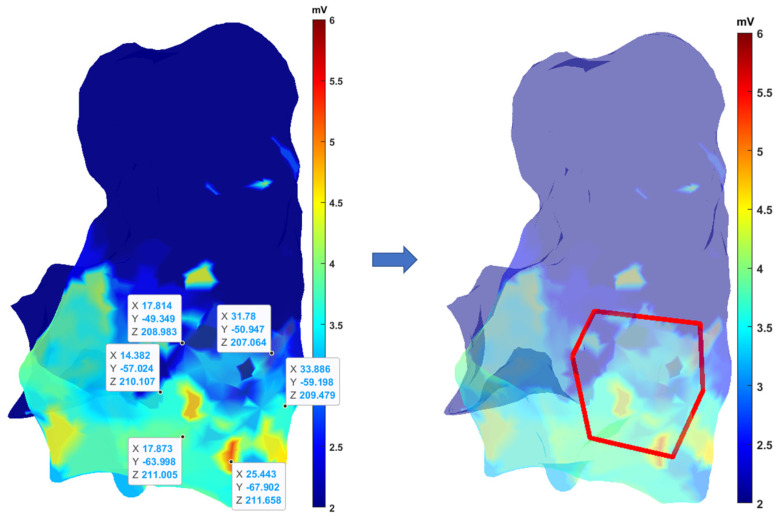
ROI selection on RV 3D map.

Algorithms have been developed to select a specific region of interest (ROI) on the RV map. This allows focused analysis of the selected area and extraction of useful information. To choose a ROI, the user must select vertices on the RV 3D map that define the ROI (Figure 3). The polygonal shape of the ROI is defined through the selection of the respective vertices. In order to avoid selecting an area where there are few electro-anatomical points, a control mesh is displayed next to it. The points selection should be made in a specific direction (clockwise or anticlockwise) in order to facilitate the generation of a correct contour. Subsequently, the selected points are stored in a data structure. Once the number of vertices to be acquired has been determined in order to provide a sufficiently precise and not overly complex outline, the coordinates of the points chosen by the newly created variable are extrapolated.

The algorithm then compares the coordinates of the electro-anatomic points with those of the selected vertices and saves only the points that fall within the ROI.

### 2.4. Multi-Parametric 3D Map

**Figure 4:**
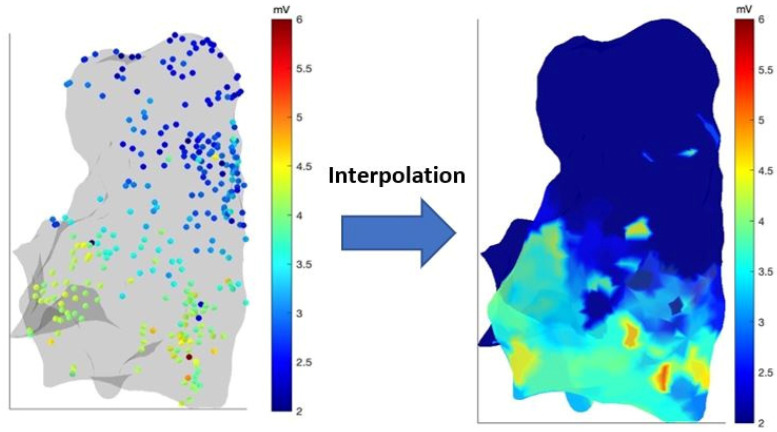
The interpolation process from scattered data to virtual surface.

A continuous, parametric color map was obtained by interpolating the scattered data of a specific feature onto the points of the virtual surface generated by anatomical mapping (Figure 4).

The sampled electro-anatomical points (temppts/tempdata) were interpolated on the points of the virtual surface generated by anatomical mapping (rPts/dataField). This enables the creation of a continuous colored parametric map, where each point of the surface (rPts) is associated with a corresponding value of the studied parameter (dataField). The OpenEP platform already provides its own function. In order to adapt the function provided by OpenEP to our purposes, we customized it by using the basic Matlab scatteredInterpolant() function, which uses a Delaunay triangulation of the scattered sample points to perform interpolation. The function accepts as input parameters both the coordinates of the acquired points (temppts) and their value (tempdata), as well as the method of interpolation and extrapolation. The term “extrapolation” refers to the estimation of an unknown value based on the extension of a known sequence of values or facts. In other words, extrapolation is inferring something that is not explicitly stated by existing information. Interpolation, on the other hand, is the act of estimating a value within two known values that exist within a sequence of values. Among the various interpolation methods available in MATLAB, the “nearest neighbor” method allows for discontinuous (discrete) interpolation, the “natural neighbor” method provides a smoother interpolation, and the “linear” method adopts a linear behavior. In this work, we utilized the “nearest neighbor” method, as we found it to be the most accurate in terms of minimum error between interpolated and measured data. The function outputs an interpolator object, which is then applied to the rPts points of the virtual surface, resulting in the vector of values associated with each of these points.

**Figure 5:**
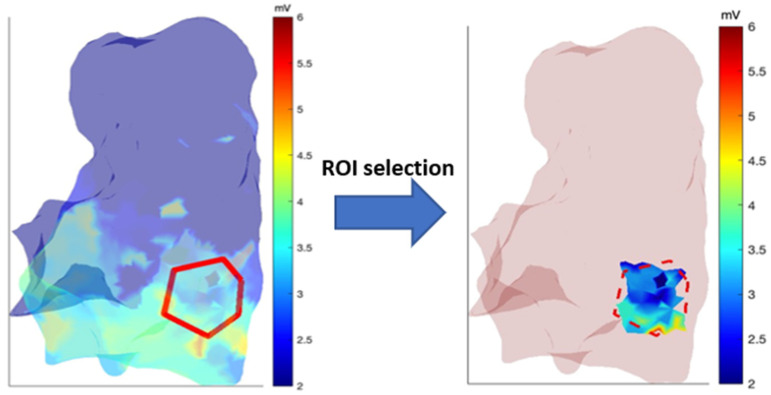
Choose of a specific ROI on the 3D parametric map and new interpolation process of data inside the ROI.

When a specific ROI is selected, as previously outlined, a new interpolation can be performed on the selected area, considering only the selected anatomical and electro-anatomical points (Figure 5).

Once a parametric 3D map is created, it may be important to evaluate the size of the RV surface where a parameter (e.g., Uni-JEl) verifies a specific condition, for example, if it is within a certain range of values. The results of this function are both numerical (e.g., size in mm^2^ or percentage of the area with respect to the RV total area) and graphical (a drawing of the specific area using a two-color scale for the 3D map). The function can also assess the size of the area where two different parameters (e.g., Uni-JEl and AT) both meet specific conditions.

#### Pre and Post Point Spatial Pairing

In the analysis of patients subjected to the ajmaline test, two different electro-anatomical acquisitions are available for the same subject, obtained in the two different phases of the test. To compare the changes of the estimated features before (pre) and after (post) the ajmaline administration, an automated algorithm was developed to identify the same RV regions in the two conditions. This is then followed by the calculation of the relative differential parameter. Due to the low half-life of the ajmaline drug (a few minutes), the number of electro-anatomical points acquired on the right ventricle surface during the post phase is typically lower than during the pre-phase. This results in a loss of points acquired during the pre-phase, where the time available to acquire electrophysiological information from the ventricle is greater. Between the two acquisitions, there is an average of 36% point loss across the entire map.

The pre and post point spatial pairing process is conducted in accordance with the following criteria: for each post point, the nearest pre point is identified utilizing the Euclidean distance between two points in space which is defined as the length of the line segment between them. The Euclidean distance between two points in Euclidean space can be calculated from their Cartesian coordinates using the Pythagorean theorem. The algorithm calculated the Euclidean distance between post and pre points to find the pre point with the minimum distance from each post point. In the following analysis, only the pair of points within the minimum distance were considered; paired points exceeding a predetermined threshold (5 mm) were excluded.

### 2.5. Data Analysis

As the final step, the algorithm produced a table that contains the numerical values of all evaluated features. This table could be used to save a single patient report or compare multiple patients and perform statistical analysis. Data analytics and statistics could be used to enable the stratification of arrhythmic risk and support a patient-specific therapeutic decision [32].

### 2.6. Application of the Workflow to Clinical Cases: Preliminary Studies

The described workflow was applied to a BRs patient (male, age: 50) who underwent an endocardial HDEAM using the CARTO system equipped with a DECANAV catheter. This subject did not show an overt type 1 ECG during the electrophysiological study and therefore underwent ajmaline challenge during HDEAM. Signals, data and maps relating to the pre-infusion phase are labelled pre and those relating to the post-infusion phase are labelled post. A 3D endocardial electro-anatomical map of the right ventricle was acquired using the CARTO 3 version 6 mapping system.

Moreover, the workflow was used to evaluate specific features in some clinical studies performed by our group. The initial application of the described workflow involved a pilot clinical study on 12 patients [33]. The aim of this study was the evaluation of the repolarization dispersion and heterogeneity. Three normal patients served as controls, while nine asymptomatic subjects with spontaneous type 1 Brugada Syndrome underwent 3D RV mapping. Using the described workflow, the ARI was calculated from the unipolar electrograms. Finally, a region of interest including the sub-pulmonary RVOT and RV free-wall was selected on the ARI 3D map. The data analysis entailed the calculation of mean ARI and ARIc (with the heart rate correction using the Bazett formula), standard deviation and interquartile range as markers of heterogeneity of dispersion. Unipolar voltage analysis was also performed with CARTO 3, setting two different voltage thresholds (≤4.4 mV and ≤5.3 mV) to detect voltage abnormalities. In another study [34] we evaluated the transmural voltage gradient dispersion and heterogeneity on two normal patients provided control data and 10 asymptomatic subjects with spontaneous type 1 BrS underwent 3D RV mapping. Among BrS patients we had 3 patients with arrhythmic events (aborted sudden death or appropriate ICD therapies) during follow-up (median 56, interquartile range: 46–74 months) and 7 patients without arrhythmic events. In the former group, we had 1 patient with inducibility of VT/VF during EPS (EPS+) and 2 patients without during EPS (EPS−); in the latter group, we had 3 patients with EPS+ and 4 patients with EPS−. Electrophysiological data and signals were treated according to the workflow described here. Uni-JEl was calculated for each point map and then interpolated to create Uni-JEl maps, interpolating data points on the mesh cell. Finally, a ROI was selected and the calculation of mean Uni-JEI (as a measure of voltage gradient dispersion), interquartile range and range (as markers of heterogeneity of dispersion) was performed. Additionally, another study [35] calculated ARI for 24 BrS patients with a persistent coved-type phenotype and correlated it with the Tpeak-Tend (Tpe) interval calculated for V1, V2 and V3 from ECG recordings, in order to investigate the relation between epicardial action potential duration and the dispersion of the repolarization measured on surface ECG in BrS patients. Finally, we applied the described workflow to investigate a population of 39 BrS subjects and 4 controls [32] in order to assess repolarization patterns and also investigate the relation between ARI, right ventricle activation time (RVAT), and Tpe in BrS patients. Patients underwent endocardial HDEAM; BrS showing an overt type 1 ECG were defined OType1, while those without (latent type 1 ECG, LType1) received ajmaline infusion. BrS patients only underwent programmed ventricular stimulation (PVS). Data were elaborated using the described workflow to obtain ARIc, while RVAT was derived from activation maps.

## 3. Results

**Figure 6:**
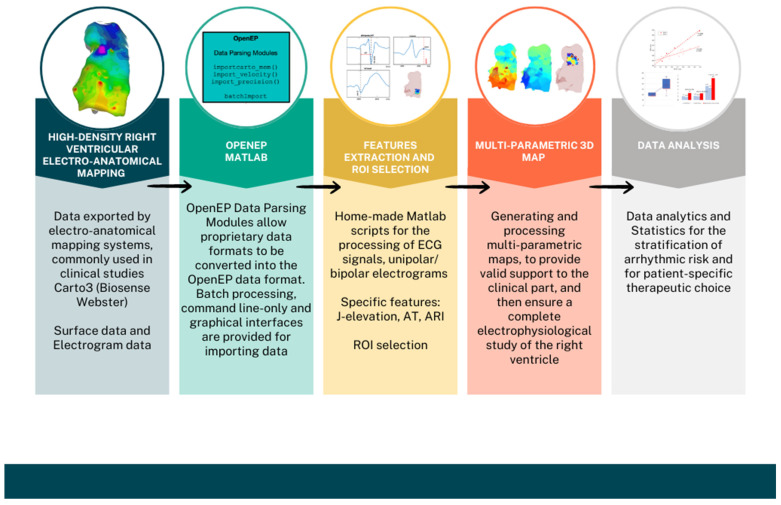
The novel workflow for electrophysiology studies in patients with Brugada Syndrome.

An overview of the described workflow is presented in Figure 6. Each block represents a procedure step, which includes the operation previously described. This section provides an example of the application of the workflow to real data, followed by a summary of clinical results recently obtained from our group.

### 3.1. Results of Real Data Application Example

**Figure 7:**
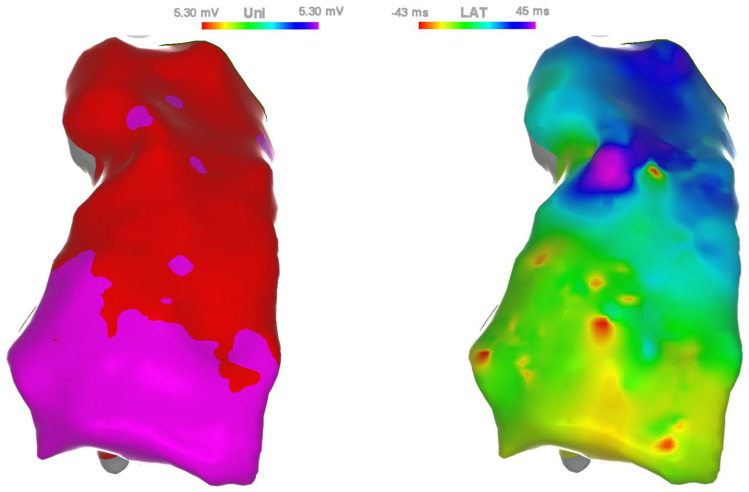
3D RV maps acquired with the CARTO system: unipolar voltages (Uni) (**left**); local activation time (LAT) (**right**).

Figure 7 shows two typical 3D RV maps, showing the unipolar voltages (Uni) (left) and the local activation time (LAT) (right), together with graphical information about the position of the heart, acquired for a BRs patient.

**Figure 8:**
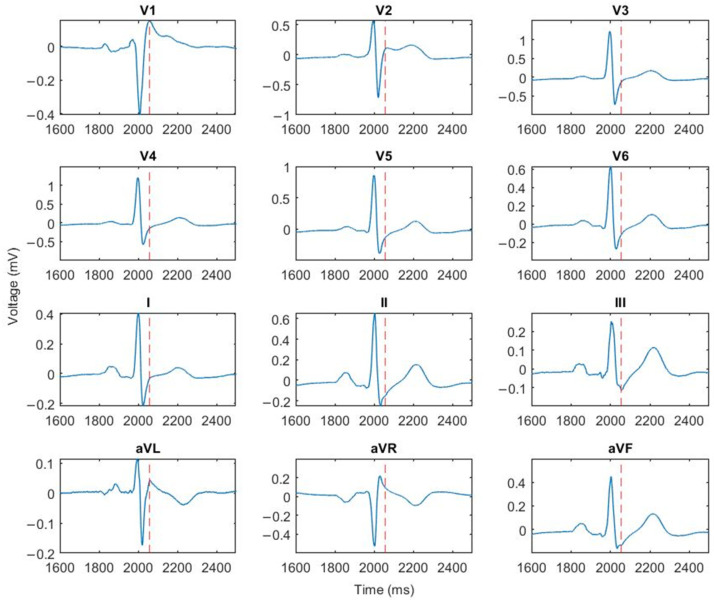
Twelve-derivation ECG and relative J-points (indicated by red dot lines).

**Figure 9:**
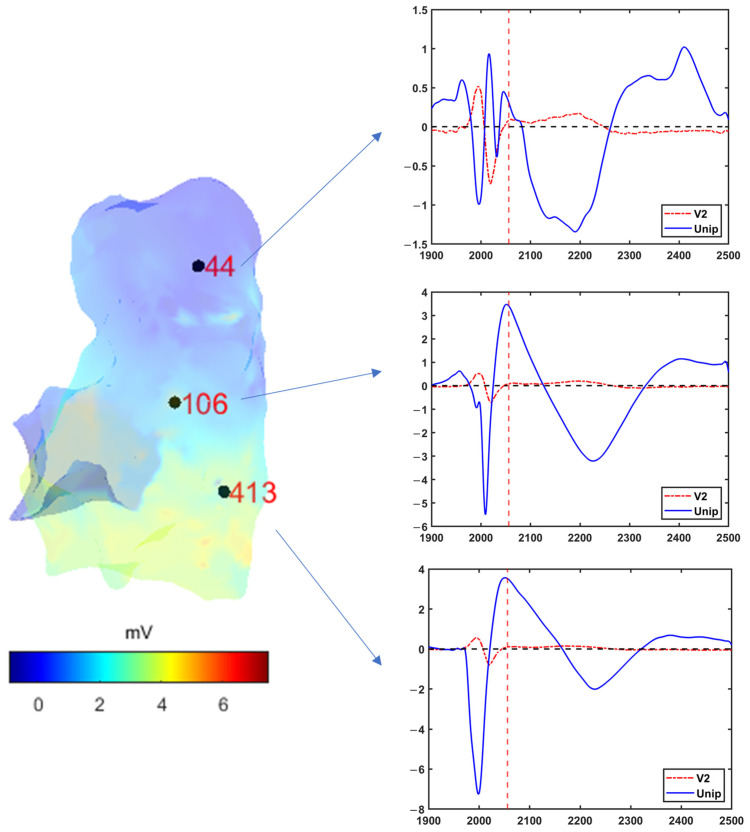
Uni-JEl 3D map (**left**); V2 ECG derivation together with unipolar signal for three specific measured locations (points #44, 106 and 413) in the RV wall (**right**).

For both the pre and post phase, the electro-anatomical information exported from the CARTO system was converted into Matlab format. The ‘userdata’ structure containing all the necessary information was created and saved. The 12 ECG derivations were then extracted from the ECG signal matrix and plotted as shown in Figure 8. The user was asked to select one ECG lead for the J-point calculation. Figure 8 shows the median values of ECG and J-point calculated from all measured points. For this patient, the V2 lead was chosen both for pre and post condition. Once the J point value has been calculated, the script calculates the Uni-JEl and produces the 3D parametric map (Figure 9, left). At this stage, one or more specific points on the map can be selected to visualize the time course of the ECG and unipolar signal at that specific position in the RV wall (Figure 9, right). This functionality allows the user to analyze and compare signals from different anatomical areas.

**Figure 10:**
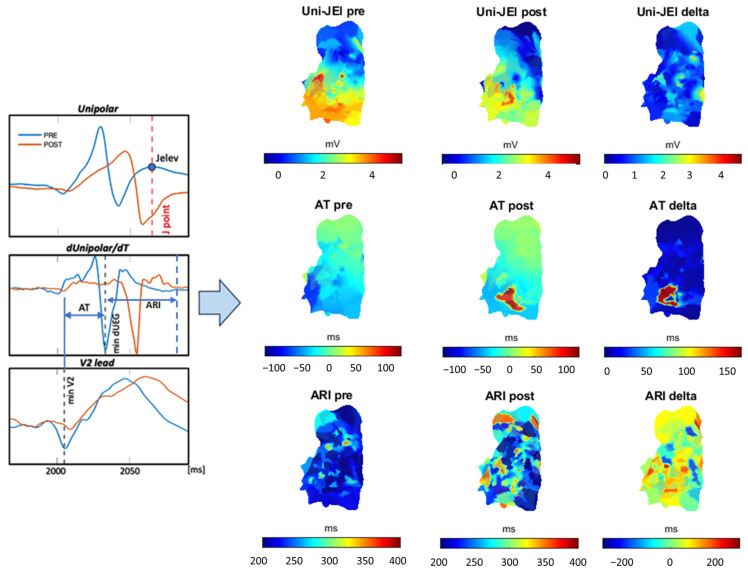
Example of one unipolar signal pair (before and after ajmaline administration) with the relative derivative over time and the corresponding V2 lead (**left**); 3D maps of the calculated features (**right**).

In order to investigate the effect of ajmaline administration on the electrophysiological characteristics (such as J-elevation of unipolar signals), the map of variation of Uni-JEl was created and shown together with the Uni-JEl *pre* and *post* maps. In addition, AT and ARI variation maps, between pre and post, were generated to assess any local depolarization and repolarization abnormalities. Figure 10 (left) shows an example of a unipolar signal pair (before and after ajmaline administration) with the relative derivative over time and the corresponding V2 lead. The plots also show how the features under consideration were calculated. The 3D maps of the calculated features are shown in Figure 10 (right). These maps were created using unipolar electrogram data after spatial registration of points obtained before and after ajmaline administration.

**Figure 11:**
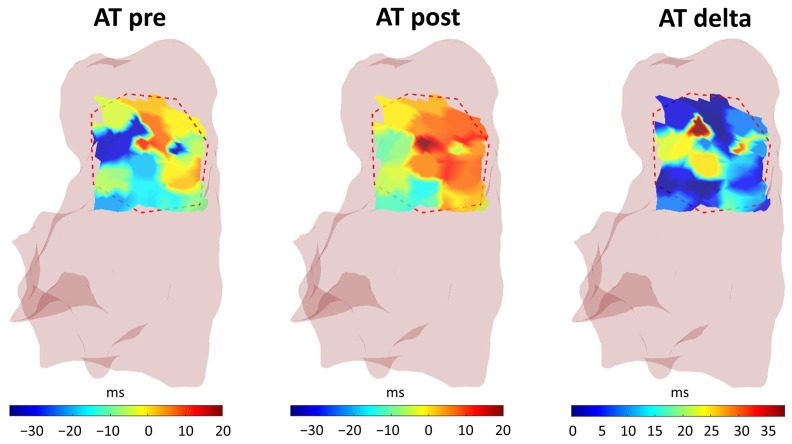
Example of a selected ROI in the RVOT region.

The ROI selection functionality was used to choose a specific region of interest in the RVOT area for better analysis of its features and variations due to the pharmacological effect. Once the user selected the ROI on the baseline 3D parametric map (before ajmaline administration), the algorithm registered the pre and post data inside the ROI, adjusted the interpolation, and calculated the parameters of interest. Figure 11 presents an example of a selected ROI in the RVOT region, for the pre, post, and variation 3D map.

**Figure 12:**
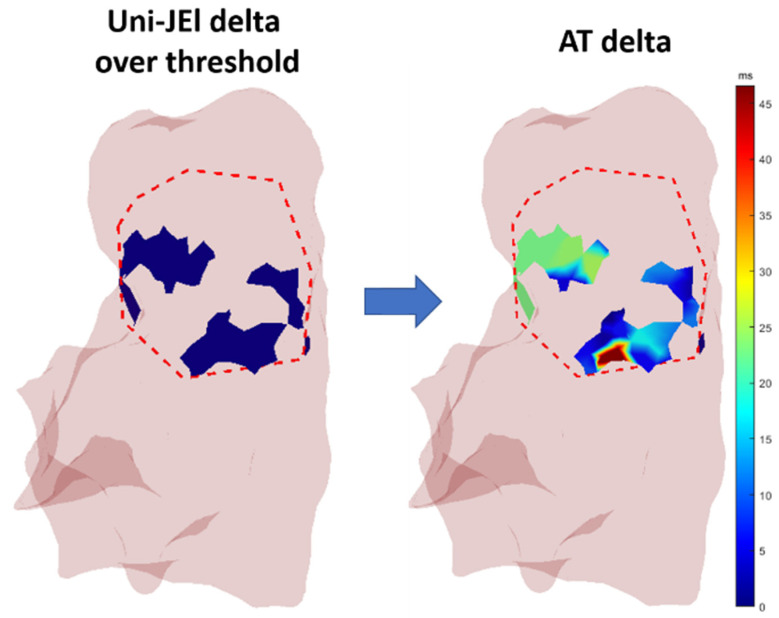
The region in which the variation of Uni-JEl exceeded a specific threshold and the variation of AT in the same region.

The algorithm calculated the area where the user-set threshold for one or more parameters was exceeded. This area was visualized in the 3D RV mesh using a two-color scale, as shown in Figure 12 for a threshold of 0.85 mV (equivalent to the median of delta Uni-JEl in the RVOT) for the Uni-JEl variations after ajmaline administration.

### 3.2. Application of the Workflow to Clinical Studies: Preliminary Results

#### 3.2.1. Repolarization Dispersion and Heterogeneity

The results of our preliminary studies demonstrated that patients with BrS exhibited greater heterogeneity of ARI dispersion, with a higher degree of dispersion compared to controls [33]. However, no differences were observed in mean ARI and mean ARIc between the two groups. Additionally, we observed a significant association between unipolar voltage abnormalities and ARI anomalies [33]. We also found that ARI well correlated with dispersion of the repolarization measured on surface ECG in BrS patients. Longer ARIc were mainly located into the anterior and sub pulmonary aspect of RVOT [35]. Finally, we also demonstrated that ARIc positively correlates with RVAT and Tpec, especially in patients showing an overt type 1 ECG (OType1) [32].

#### 3.2.2. Transmural Voltage Gradient Dispersion and Heterogeneity

Results of our study showed [34] that BrS patients had Muni-JEl, intrqUni-JEl and ΔUni-JEI higher than controls. Morover, BrS patients with arrhythmic events during the follow-up showed higher intrqUni-JEl and the ΔUni-JEI respect to BrS with EPS+ and without arrhythmic events during follow-up.

## 4. Discussion

This work presents an innovative data processing workflow for endocardial signals acquired with HDEAM systems from BrS patients, calculating the parameters of interest and creating multi-parametric maps of the RV. The workflow allows for a thorough analysis of the electrophysiological condition of the right ventricle which can be useful for both research purposes and for determining a suitable patient-specific treatment plan.

It is based on the OpenEP framework, entirely using Matlab software and available under an open-source license. OpenEP has already been validated [25] and is widely used in the literature [36,37,38,39,40,41,42,43]. The OpenEP framework includes functionalities such as analysis of chamber geometry, activation mapping, conduction velocity mapping, voltage mapping, ablation sites, and electrograms, as well as visualization and input/output functions. However, contemporary studies with clinical or research purposes may require additional functionalities. Our workflow allows the calculation of certain features, such as unipolar elevation at J-point, activation time, and activation recovery interval, across the entire RV area or in specific regions of interest, such as RVOT. These parameters can then be visualized on a 3D parametric map. In this paper we provide a technical description of the device’s functionalities, along with the results obtained on a patient with BrS who underwent an endocardial HDEAM. Such workflow has already been implemented by our study group in clinical research projects carried out on a population of asymptomatic Brugada patients [32,33,34,35,44].

The workflow is designed in order to enable numerical analysis even in large patient populations of multi-center studies, guaranteeing homogeneity even in larger cohorts. This might also support the application of artificial intelligence methods in this field of study. The development and optimization of the workflow require an interdisciplinary group comprising of physicians, clinicians, biomedical engineers, software and product engineers. Further developments could include the analysis of both endocardial and epicardial signals, acquired, for example, using Noninvasive Electrocardiographic Imaging (ECGI) [45], to better characterize the electrical substrate of asymptomatic BrS subjects.

In conclusion, as supported by previous studies performed by our group, the presented workflow can be useful in evaluating multi-parametric maps in an automatic and standardized manner through high-density point-by-point analysis of endocardial signals in a BrS population. In our experience, (1) it was versatile and easy to apply in a clinical research context; (2) it facilitated the performance of patient-specific data analytics and statistics; (3) it was able to adjust to the innovations and modifications of electrophysiological studies.

## Data Availability

The datasets presented in this article are not readily available because the data are part of an ongoing study.
